# Potential Hepatoprotective Effects of Allicin on Carbon Tetrachloride-Induced Acute Liver Injury in Mice by Inhibiting Oxidative Stress, Inflammation, and Apoptosis

**DOI:** 10.3390/toxics12050328

**Published:** 2024-04-30

**Authors:** Qianmei Gong, Xiaoming Wang, Yongshi Liu, Heling Yuan, Zifeng Ge, Yuzhou Li, Jinhu Huang, Yufan Liu, Ming Chen, Wenjun Xiao, Ruiting Liu, Rongmei Shi, Liping Wang

**Affiliations:** 1 MOE Joint International Research Laboratory of Animal Health and Food Safety, College of Veterinary Medicine, Nanjing Agricultural University, Nanjing 210095, China; 2 College of Pharmacy, Xinjiang Medical University, Urumqi 830017, China

**Keywords:** allicin, acute liver injury, anti-inflammation, proinflammatory cytokine, Nrf2

## Abstract

The global burden of liver disease is enormous, which highlights the need for effective hepatoprotective agents. It was reported that allicin exhibits protective effects against a range of diseases. In this study, we further evaluated allicin’s effect and mechanism in acute hepatic injury. Liver injury in mice was induced by intraperitoneal injection with 1% CCl_4_ (10 mL/kg/day). When the first dose was given, CCl_4_ was given immediately after administration of different doses of allicin (40, 20, and 10 mg/kg/day) as well as compound glycyrrhizin (CGI, 80 mg/kg/day), and then different doses of allicin (40, 20, and 10 mg/kg/day) as well as compound glycyrrhizin (CGI, 80 mg/kg/day) were administrated every 12 h. The animals were dissected 24 h after the first administration. The findings demonstrated a significant inhibition of CCl_4_-induced acute liver injury following allicin treatment. This inhibition was evidenced by notable reductions in serum levels of transaminases, specifically aspartate transaminase, along with mitigated histological damage to the liver. In this protective process, allicin plays the role of reducing the amounts or the expression levels of proinflammatory cytokines, IL-1β, IL-6. Furthermore, allicin recovered the activities of the antioxidant enzyme catalase (CAT) and reduced the production of malondialdehyde (MDA) in a dose-dependent manner, and also reduced liver Caspase 3, Caspase 8, and BAX to inhibit liver cell apoptosis. Further analysis showed that the administration of allicin inhibited the increased protein levels of Nuclear factor-erythroid 2-related factor 2 (Nrf2) and NAD(P)H:quinone oxidoreductase 1 (NQO1), which is related to inflammation and oxidative stress. The in vitro study of the LPS-induced RAW264.7 inflammatory cell model confirmed that allicin can inhibit important inflammation-related factors and alleviate inflammation. This research firstly clarified that allicin has a significant protective effect on CCl_4_-induced liver injury via inhibiting the inflammatory response and hepatocyte apoptosis, alleviating oxidative stress associated with the progress of liver damage, highlighting the potential of allicin as a hepatoprotective agent.

## 1. Introduction

Liver disease remains a significant global burden with regard to mortality rates and associated costs. The disease’s high morbidity and mortality levels contribute to its continued impact on global health [1]. It is becoming a major concern in terms of global public health. It is estimated that more than 10% of the worldwide population suffers from this condition. In China, the annual incidence of liver injury caused by medication alone is estimated to be 23.80 cases per 100,000 individuals [2]. In addition, it also severely affects the health of animals. Liver damage can be triggered by a variety of factors, such as drug irritations, viral infections, alcohol, chemical poisons, or obesity [3,4]. Numerous studies have demonstrated that liver injury is triggered by two primary pathological processes: inflammation and oxidative stress [5,6]. Therefore, it is evident that mitigating inflammation and oxidative stress plays a crucial role in alleviating acute liver injury. Liver disease poses a significant threat to both human and animal health. This highlights an urgent need to explore safe and effective “off-the-shelf” drugs for treatment. Many synthetic drugs have been found to have negative impacts on the liver. As a result, there is now significant focus on complementary and alternative medicines for the treatment of hepatic disorders.

Since antiquity, garlic has been utilized globally as a flavor enhancer and herb, holding particular significance globally, especially in China and other regions of the world. Garlic has become a prevalent ingredient in modern cuisine and has generated a lucrative market for health supplements worth millions of pounds. Allicin constitutes the principal functional constituent of raw garlic puree. It emerges from the transformation of alliin into alliinase via the action of alliin, which is caused when the clove is crushed [7]. Garlic exhibits a variety of pharmacological properties, including antimicrobial effects, the inhibition of inflammatory response, and the scavenging of oxygen free radicals [8,9]. Allicin has been found to ameliorate intervertebral disc degeneration through the inhibition of oxidative stress and reduction in apoptosis [10], and it can also delay the progression of pulmonary arterial hypertension through the modulation of proinflammatory and profibrotic markers in the lung and heart [11]. Additionally, certain studies have indicated that allicin has the potential to reduce hepatic steatosis and enhance cardiovascular phenotypes through the modulation of gut microbiota [12,13,14]. Importantly, Zhao et al. [15] recently reported that H2S-mediated Keap1 S-sulfhydration alleviates liver damage through the activation of Nrf2 and that the administration of the exogenous H2S donor plays a protective role in streptozotocin (STZ) plus HFD-induced, or CCl_4_-stimulated, liver dysfunction. Dong et al. [16] found that by activating Nrf2, the levels of TNF-α and IL-1β in the hippocampus can be significantly reduced, thereby alleviating oxidative stress injury in the hippocampus of rats. Ma et al. [17] found inhibited Toll-like receptor 2 (TLR2) and Toll-like receptor 4 (TLR4) activation and mitogen-activated protein kinase (MAPK) phosphorylation, which in turn inactivated the inflammatory cytokines in the livers of the CCl_4_-treated mice. Our previous pilot study discovered that allicin can be converted to H2S upon administration to rats. (Data not published). Therefore, in the present study, we investigated the potential protective effect of allicin pretreatment on LPS-induced inflammation in a cell model and CCl_4_-induced acute liver injury in a murine model.

## 2. Results

### 2.1. Allicin Pretreatment Inhibits NO Production and Cytokine Expression in LPS-Stimulated RAW264.7 Cells

**Figure 1:**
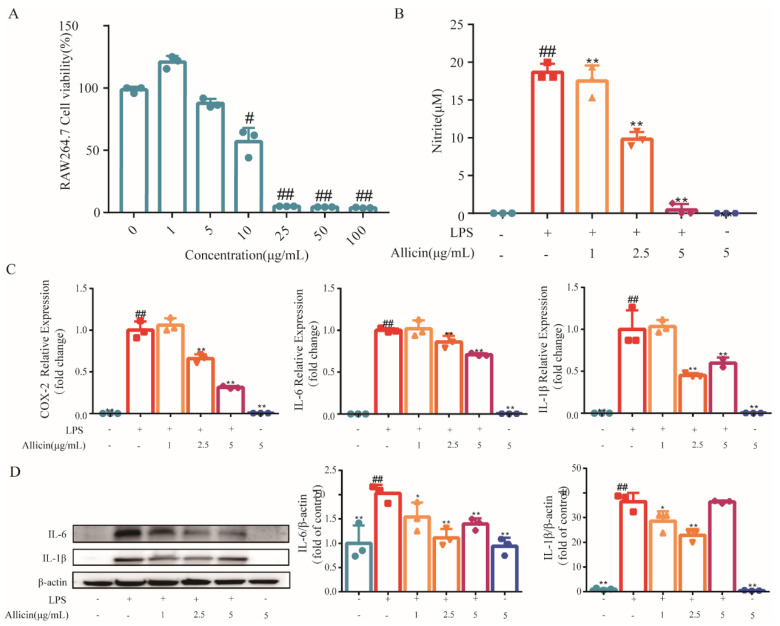
Allicin pretreatment inhibits NO production and cytokine expression in LPS-stimulated RAW264.7 cells. (**A**) The cytotoxicity of allicin in RAW264.7 cells was determined by CCK-8 assay after 24 h treatment. (**B**) RAW264.7 cells treated with allicin (1, 2.5, 5 μg/mL) for 9 h and then induced with LPS (1 μg/mL) for 18 h. (**C**) The effect of allicin on the expression of COX-2, IL-6, and IL-1β mRNA in RAW264.7 cells. (**D**) The IL-6 and IL-1β expression proteins were detected using Western blotting. Significant vs. untreated control, ## *p* < 0.01, # *p* < 0.05; significant vs. LPS treated cell, ** *p* < 0.01, * *p* < 0.05.

Figure 1A demonstrates that allicin has no effect on cell viability within the concentration range of 1−5 μg/mL when treated for 24 h. Therefore, we chose 1, 2.5, and 5 μg/mL to treat the cells. We found that the production of NO in the allicin pretreatment groups was obviously decreased with a dose-dependent manner (*p* < 0.01) compared with the LPS group (Figure 1B). Considering that LPS accelerates inflammatory responses via the production of proinflammatory cytokines in macrophages, we further detected the effect of allicin on the expression of COX-2, IL-6, and IL-1β in RAW264.7 cells using qRT-PCR and Western blot. The results showed that allicin at the concentration of 2.5 and 5 ug/mL could significantly inhibit the mRNA expression of COX-2, IL-6, and IL-1β (*p* < 0.01) (Figure 1C). Protein expression levels of IL-6 and IL-1β were significantly inhibited in RAW264.7 cells after pretreatment with allicin (*p* < 0.05) (Figure 1D). The results indicated that allicin had a protective effect on LPS-induced inflammation in RAW264.7 cells.

### 2.2. Allicin Suppressed ROS Generation in LPS-Stimulated RAW264.7 Cells

**Figure 2:**
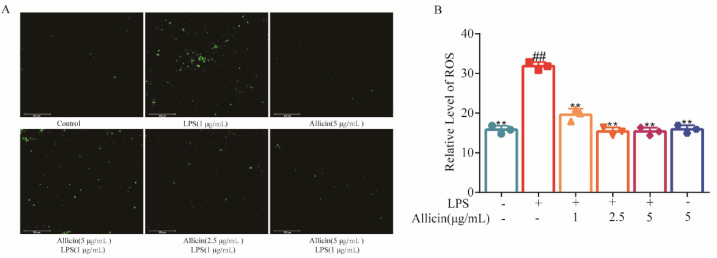
Allicin inhibited ROS generation in LPS-stimulated RAW264.7 cells. (**A**) ROS level in cells was stained with DCFH2-DA. The images were captured by fluorescence microscopy. (**B**) Relative levels of ROS reflected by fluorescence intensity. Significant vs. untreated control, ## *p* < 0.01; significant vs. LPS treated cell, ** *p* < 0.01.

Compared with the blank control group, the ROS content of the cells in the LPS group significantly increased (*p* < 0.01). Pretreatment of the RAW264.7 cells with high, medium, and low doses of allicin for 9 h can significantly inhibit the generation of ROS in the cells in a dose-dependent manner (*p* < 0.01), indicating that allicin can inhibit LPS-induced ROS generation in RAW264.7 cells (Figure 2). 

### 2.3. Allicin Pretreatment Attenuates CCl_4_-Induced Acute Liver Injury in Mice

**Figure 3:**
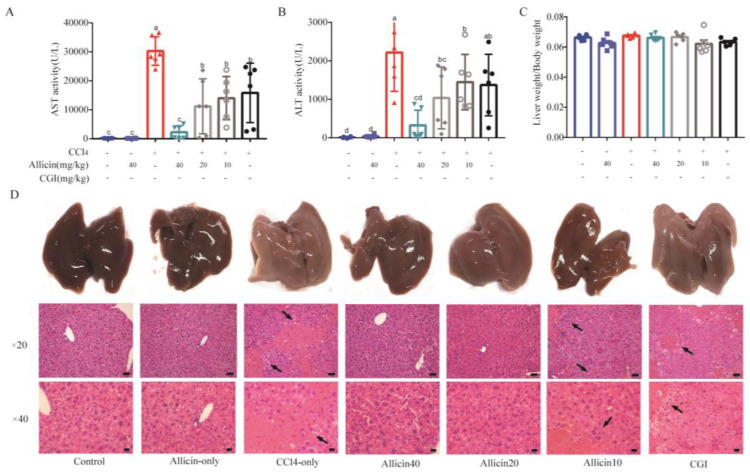
Allicin alleviated CCl_4_-induced acute liver injury in mice. (**A**) The inhibitory effects of Sal on serum aspartate aminotransferase (AST) (**B**) The inhibitory effects of Sal on alanine aminotransferase (ALT). (**C**,**D**) Effects of allicin on histopathological changes in the liver in mice with CCl_4_ acute liver injury liver (Bar = 50 μm).

As shown in Figure 3A,B, the levels of serum AST and ALT were markedly elevated in the CCl_4_ control group compared with the control group (*p* < 0.01), indicating that the model of acute liver injury induced by CCl_4_ was successfully established. However, allicin significantly decreased the serum AST and ALT in a dose-dependent manner when compared with the CCl_4_ control group (*p* < 0.05). In addition, the level of AST and ALT in the allicin high-dose group was even obviously lower than that of the CGI group (*p* < 0.05), which is widely prescribed for treating hepatic injury. Histological changes are the gold standard for evaluating hepatic tissue injury. Although the liver/body ratios were not changed among the different groups, compared with the normal group, CCl_4_ induced a severe destruction of hepatic architecture with various degrees of hepatocyte necrosis and inflammatory cell infiltration, while allicin improved the morphology of the liver, the degree of inflammation, and the size of hepatocyte necrosis in a dose-dependent manner (Figure 3C,D).

### 2.4. Allicin Alleviated CCl_4_-Induced Hepatocytes Apoptosis

**Figure 4:**
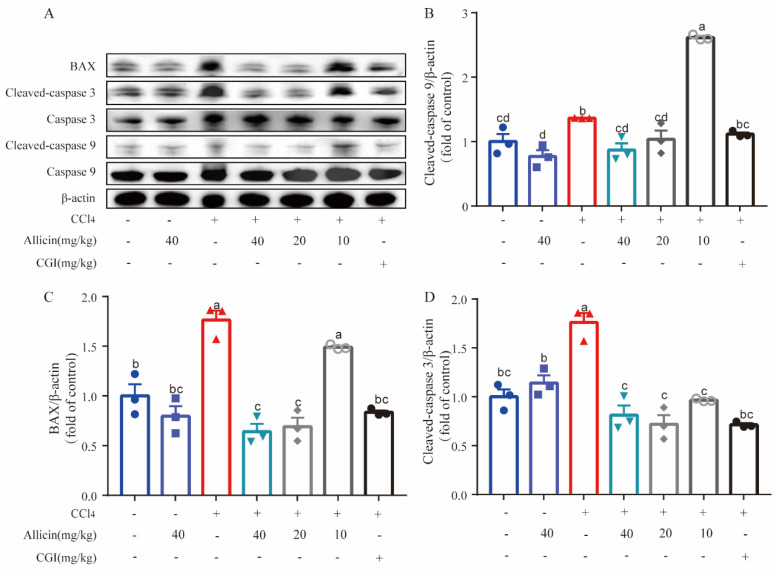
Allicin inhibited CCl_4_-induced apoptosis in mice. (**A**) The BAX, Caspase 3, and Caspase 9 expression proteins were detected using Western blotting. (**B**) Statistical analysis of the protein expression of BAX. (**C**) Statistical analysis of the protein expression of Caspase 3. (**D**) Statistical analysis of the protein expression of Caspase 9. Values represent the mean ± SD (n = 6).

The levels of Caspase 3, Caspase 9, and Bax protein expression in the liver tissues were detected by Western blot to assess apoptosis. In comparison, the expression levels of cleaved Caspase 3, cleaved Caspase 9, and Bax protein in the CCl_4_ model group were significantly higher than those of the normal control (*p* < 0.01), indicating that CCl_4_ could induce hepatocyte apoptosis. Allicin dose-dependently downregulated cleaved Caspase 3 expression in the liver when compared with the CCl_4_ model group (*p* < 0.05). High and medium doses of allicin (40 and 20 mg/kg) significantly reduced the expression of cleaved Caspase 9 and BAX in the liver tissue of mice (*p* < 0.05); the low dose of allicin had no significant effect on the expression of cleaved Caspase 9 and BAX protein (Figure 4). CGI also significantly decreased the level of cleaved Caspase 9 and BAX proteins (*p* < 0.05).

### 2.5. Allicin Suppressed CCl_4_-Induced Hepatic Inflammatory Response

**Figure 5:**
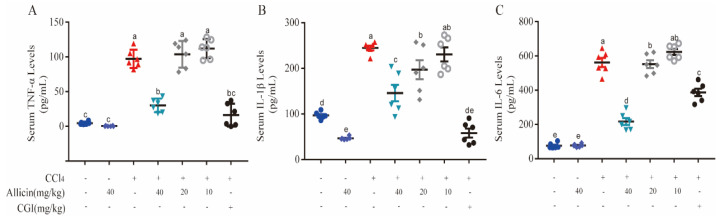
Allicin suppressed CCl_4_-induced inflammatory cytokines in mice. (**A**) Hepatic TNF-a, protein levels were detected by ELISA. (**B**) Hepatic IL-1β protein levels were detected by ELISA. (**C**) Hepatic IL-6 protein levels were detected by ELISA. Values represent the mean ± SD (n = 6).

Inflammatory responses play a pivotal role in inducing liver injury. In this study, we assessed the release of three critical proinflammatory cytokines, TNF-α, IL-1β, and IL-6, in the serum using ELISA kits. As shown in Figure 5, the levels of TNF-α, IL-1β, and IL-6 in the CCl_4_ group were significantly higher than those in the normal control group and the allicin control group (*p* < 0.01), indicating that CCl_4_ caused a large amount of inflammatory factors release. Allicin with a high dose (40 mg/kg) could significantly reduce serum TNF-α, IL-1β, and IL-6 levels in mice (*p* < 0.01), with a medium dose (20 mg/kg) only obviously reducing the IL-1β level (*p* < 0.05). However, CGI can significantly reduce the serum content of TNF-α, IL-1β, and IL-6 in mice (*p* < 0.05), showing a better anti-inflammatory effect.

### 2.6. Allicin Attenuates CCl_4_-Induced Oxidative Stress in Mice

**Figure 6:**
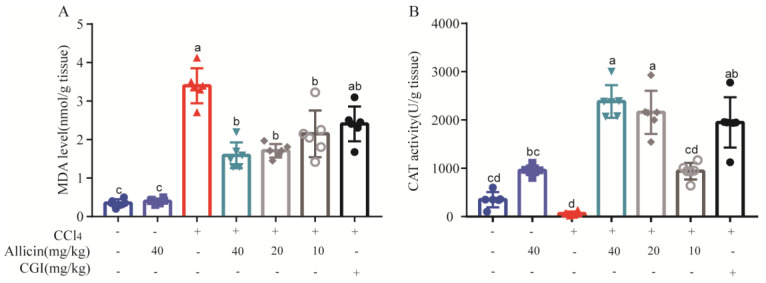
Allicin modulated CCl_4_-induced oxidative stress in mice. (**A**) Hepatic MDA content. (**B**) Hepatic CAT activity. Values represent the mean ± SD (n = 6).

Based on the association of oxidative stress markers with hepatic injury following CCl_4_ induction, we further measured MDA and CAT levels. As illustrated in Figure 6, CCl_4_ resulted in a significant increase in hepatic MDA content and a decrease in CAT level relative to the normal control (*p* < 0.01). Notably, allicin dose-dependently inhibited MDA release and increased the activity of CAT (*p* < 0.01). CGI could also significantly increase the content of CAT in the liver (*p* < 0.01); however, there was no obvious effect on MDA level. This indicated that allicin as well as CGI had an antioxidant activity and relieved the liver oxidative stress induced by CCl_4_.

### 2.7. Allicin Activates Nrf2/NQO1 Pathway following CCl_4_-Induced Acute Liver Injury

**Figure 7:**
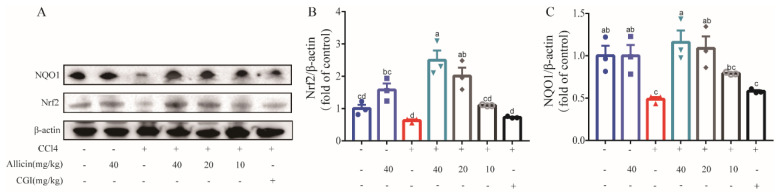
Allicin upregulated the levels of hepatic Nrf2 and NQO1 protein expression in mice. (**A**) The Nrf2 and NQO_1_ expression proteins were detected using Western blotting. (**B**) Statistical analysis of the protein expression of Nrf2. (**C**) Statistical analysis of the protein expression of NQO1.Values represent the mean ± SD (n = 6).

Having found that allicin attenuates CCl_4_-induced oxidative stress, we also investigated the pathway involved in the hepatoprotective effect of allicin. Nrf2 is considered critical for the activation of antioxidant activity. Therefore, we detected the expression of Nrf2 and its downstream target NAD(P)H:quinone oxidoreductase 1 (NQO1) protein in mouse liver tissue by Western blotting. As shown in Figure 7, the Nrf2 protein level was obviously downregulated in the CCl_4_ group compared with the normal control group (*p* < 0.01). However, allicin at 40 and 20 mg/kg markedly increased the levels of Nrf2 in the nucleoprotein (*p* < 0.01); the 10 mg/kg group showed a slightly higher expression but with no statistical significance (Figure 7A,B). In line with the above results, the expression of NQO1 proteins showed a similar tendency. Nevertheless, CGI did not show an obvious effect on the expression of Nrf2 and NQO1. Together, these data indicated that allicin attenuated CCl_4_-induced liver injury via nuclear Nrf2 activation and upregulation of the Nrf2 downstream target NQO1. This highlights the clinical potential of allicin as a promising therapeutic strategy for managing acute liver injury.

## 3. Discussion

Liver diseases pose a global health concern, and currently available medical interventions are often inadequately effective. In recent years, extensive research has indicated that natural products offer hepatoprotective benefits through their various mechanisms of action in oxidant/antioxidant balance, inflammation, apoptosis, and damage responses. It is commonly acknowledged that allicin is linked to numerous health advantages in humans such as the decreased probability of developing diverse cancers, especially in the gastrointestinal tract [18], as well as cardiovascular disease [19] and type 2 diabetes [20]. In this study, the objective was to investigate the hepatoprotective impact and associated mechanisms of natural allicin, which is a leading bioactive compound derived from garlic. We provide demonstrable evidence that allicin effectively attenuates CCl_4_-induced acute liver damage and enhances liver function in mice.

CCl_4_-induced acute liver injury in mice has been widely used as a classical animal model to evaluate the hepatotoxicity of developed drugs [21]. CCl_4_ could activate the liver microsomal cytochrome P450 to produce CCl_3_ and CCl_3_OO. Free radicals can react with macromolecules such as lipids, proteins, and nucleic acids in hepatocytes, causing lipid peroxidation and subsequently damaging the stability and integrity of various biological membranes. In the course of liver injury caused by CCl_4_, AST, ALT, and ALP are passively released into the extracellular milieu by dying or injured liver cells and enter into blood; therefore, serum AST, ALT, and ALP are commonly used as indicators for evaluating liver injury [22,23]. In this study, it is demonstrated that the levels of AST, ALT, and ALP in the serum are associated with the extent of histopathological damage found in the livers of mice treated with CCl_4_, and that allicin treatment can mitigate this effect in a dose-dependent manner.

Two pathological events, namely oxidative stress and inflammation, are examined in this investigation to reveal the mechanism underlying the hepatoprotective effect of allicin [6,24]. We initially investigated the anti-inflammatory impact of allicin by utilizing a LPS-induced cell model and CCl_4_-induced liver injury in mice. Our findings revealed that allicin significantly restrained the classic inflammatory mediators, such as IL-6 and IL-1β, in both mouse RAW264.7 macrophages and serum samples, indicating a robust anti-inflammatory effect of allicin. Alternatively, some researchers regard that garlic being protective across such a wide spectrum of diseases is related to garlic’s ability to alter gaseous signaling molecules like NO generated by the L-arginine-NO synthase (NOS) pathway by three NOS enzymes in mammals [25,26]. NO has been shown to play significant roles in inflammation, infection, and diabetes [27]. In this study, it was demonstrated that allicin, the primary component of garlic, can also suppress the production of NO in RAW264.7 mouse macrophages stimulated with LPS. This may lead to a synergistic inhibition of classic inflammatory factors, resulting in positive anti-inflammatory effects. Our findings provide evidence to support previous studies that suggest a link between the anti-inflammatory properties of garlic and the inhibition of NO production [28,29,30,31]. The precise process of allicin altering gas signaling molecular NO deserves further investigation, which will be helpful to clarify the mechanism of garlic being protective across such a wide spectrum of diseases.

It is well known that oxidative stress caused by reactive oxygen species (ROS) like CCl_3_· and CCl_3_OO· during damage results in hepatocellular inflammation, apoptosis, and necrosis, and that the detoxifying system is essential for preventing ROS injury via the elimination of free radicals [32]. In this study, we found allicin could reduce ROS and MDA (the metabolic end product of lipid peroxidation, which can reflect the lipid peroxidation in animals and can induce cell damage [33,34]) levels while increasing CAT (the important endogenous antioxidants in animals) activity in cells or mice, which is similar to the effect of allicin in a mouse model of hepatic I/R injury [35]. As anticipated, our findings demonstrate that treatment with CCl_4_ leads to a noteworthy upsurge in the expression of Caspase 9, Caspase 3, and BAX in the liver tissue. This indicates that CCl_4_ instigates the apoptosis of hepatocytes. Compared with the CCl_4_ group, the protein expressions of Caspase 9, Caspase 3, and BAX in the high- and medium-dose allicin groups were significantly decreased, which proved that allicin plays a protective role in liver injury through anti-apoptotic effects. Apoptosis represents the initial reaction of hepatocytes to various damaging factors. Subsequently, necrosis commonly follows apoptosis, and the apoptotic process manifests as a critical factor in hepatocyte necrosis development [36,37]. Our results indicate allicin could protect cells from apoptosis to prevent progress to necrosis.

The Nrf2 signaling pathway boasts significant importance in terms of the body’s response to oxidative stress-related damage. It has the potential to elevate the level of antioxidants by upregulating the antioxidant proteins present in liver cells [38]. Under a normal physiological state, Nrf2 and its suppressor Keap1 form an inactive complex in the cytoplasm; however, when hepatocytes are stimulated by oxidative substances, Nrf2 dissociates from Keap1 and transfers into the nucleus to promote the expression of its downstream proteins like NQO1, and, accordingly, to reduce the level of free radicals in the body [39]. The research illustrates that allicin can substantially upregulate the protein expression of both Nrf2 and NQO1 in the liver tissue whilst also modulating the Nrf2 signaling pathway. Moreover, it upgrades the body’s antioxidant potential and aids in shielding the liver against CCl_4_-induced hepatic oxidative stress as well as its associated signaling activities.

**Figure 8:**
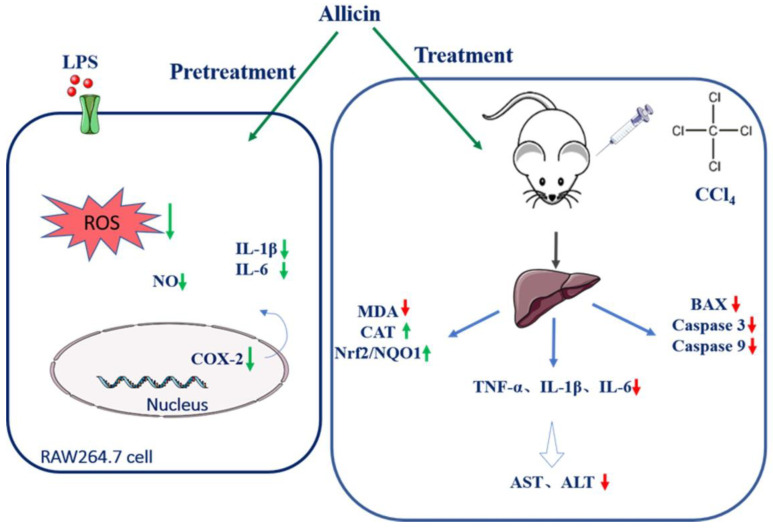
Schematic diagram showing that allicin administration suppressed CCl_4_-mediated hepatotoxicity through activation of the Nrf2 signaling pathway and downstream antioxidant genes that attenuate oxidative insult and inflammatory and apoptotic responses.

In addition, CGI have been proved to have anti-inflammatory, immune regulation, and other effects, including promoting liver cell proliferation, and have been widely used in clinical practice at this stage [40]. According to our results, allicin could be comparable to the anti-inflammatory and hepatoprotective effects of drug CGI. Taken together, our data indicate that allicin has significant protective effects against CCl_4_-induced liver injury by reducing hepatocyte apoptosis, inhibiting oxidative stress, and reducing inflammation through the regulation of the Nrf2/NQO1 pathway (Figure 8). Our study indicates that the impact of allicin or garlic on health and disease processes may be far more complex than initially believed, and their overall role in promoting contributions to health may have been potentially underestimated.

## 4. Materials and Methods

### 4.1. Reagents and Antibodies

Alliin (purity 84%) and aliases (titer > 10,000 IU/g) were obtained from Xinjiang Medical University. CCl_4_ was purchased from Wanqinghua Glass Instrument Co., Ltd. (Nanjing, China). Primary antibodies against β-actin, IL-1β, IL-6, Keap1, and Nrf-2 were purchased from Abcam (Cambridge, MA, USA); Caspase 3, Caspase 9, and BAX monoclonal antibodies were purchased from Abmart (Shanghai, China); compound glycyrrhizin (CGI) tablets (Ganyu, China) (specification: glycyrrhizin 25 mg, glycyrrhizic acid 25 mg, DL-methionine 25 mg) were purchased from Kain Technology Co., Ltd. (Beijing, China).

### 4.2. Allicin Preparation and Content Quantification

**Figure 9:**
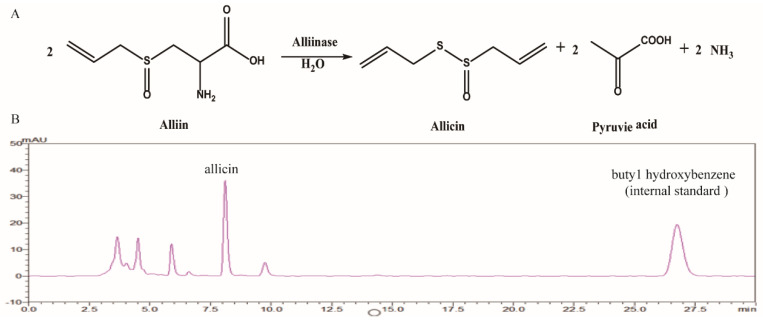
(**A**) Formation of allicin from alliin, which is catalyzed using alliinase enzyme. (**B**) Conversion of allicin from alliin and detection of allicin by HPLC.

Alliinase catalyzes the conversion of alliin (1 g alliin) to allicin (0.458 g) and the concentrations of allicin stock solution (1 mL/mg) produced by alliin (21.82 mg) were rechecked by HPLC as previously described [41] in this study and used to conduct the cell-based assays and animal studies (Figure 9A,B).

### 4.3. Cell Culture

RAW264.7 cells, a line of mouse macrophage, were purchased from the cell bank of the type culture collection committee of the Chinese Academy of Sciences, and cultured in DMEM (Gibco) containing 10% fetal bovine serum (Zeta) in a 5% CO_2_ incubator at 37 °C.

### 4.4. LPS-Induced Inflammation in RAW264.7 Cells and Allicin Treatment

#### 4.4.1. Cell Groups and Treatment

**Figure 10:**
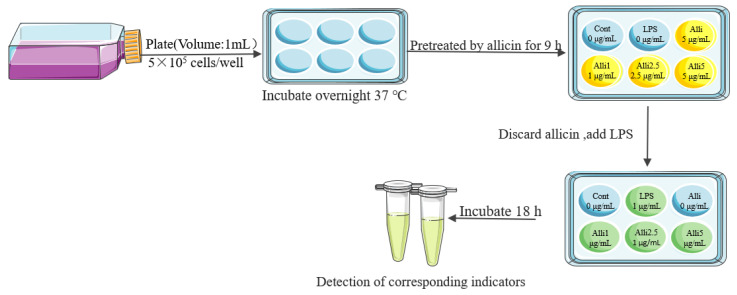
Cell processing process.

RAW264.7 cells were plated and then divided into 6 groups: normal cell control (NC group, DMEM), allicin control (AC group), LPS control (LPS group, 1 μg/mL), and treatment groups of allicin with high (AH group, 5 μg/mL), medium (AM group, 2.5 μg/mL), and low doses (AL group, 1 μg/mL). In all allicin treatment groups, the cells were pretreated by allicin (5, 2.5 and 1 μg/mL) for 9 h and the cells were washed to discard the allicin; then, 1 μg/mL LPS was added in cell culture for 18 h and the samples were collected according to the detected indicators (Figure 10).

#### 4.4.2. Detection of NO Production in RAW264.7 Cells

The macrophages were treated with allicin followed by LPS stimulation as described above. NO formation in the cultured medium was measured by mixing a 50 μL cultured medium and a Griess reagent. The absorbance at 540 nm was measured. The NO concentration was calculated using the sodium nitrite standard curve.

#### 4.4.3. RNA Isolation and Real-Time Quantitative PCR (Real-Time RT-PCR)

In order to investigate the mRNA expression levels of COX-2 (cyclooxygenase-2), IL-6 (interleukin 6) and IL-1β (interleukin 1 beta) total RNA was isolated from cells treated with allicin (1, 2.5, or 5 μg/mL) or LPS (1 μg/mL) for 12 h and reverse-transcribed to cDNA using HiScript III RT SuperMix kit (Vazyme, Nanjing, China) based on the manufacturer’s protocol. Then, the COX-2, IL-1β, IL-6, and β-actin cDNA was amplified by ChamQ SYBR Color Qpcr Master Mix (Vazyme, Nanjing, China) using the following conditions: 1 cycle for 180 s at 95 °C; 26 cycles for 55 s at 93 °C, 45 s at 60 °C, and 40 s at 72 °C; and 1 cycle for 100 s at 72 °C. The primers used in this experiment are presented in Table 1. All reactions were conducted in triplicate. β-actin was used as an internal control, and the fold change in gene expression was calculated using the 2^−ΔΔCT^ method.

#### 4.4.4. Western Blot Analysis for IL-6 and IL-1β Expression

The total proteins from RAW264.7 cells were extracted using Radio Immunoprecipitation Assay (RIPA) lysis solution (Beyotime, Shanghai, China) containing phenylmethanesulfonyl fluoride (PMSF) on ice, following the manufacturer’s instructions. Protein quantification was conducted using the BCA protein content determination method. For Western blotting analysis, an equal amount of protein (40 µg) was utilized, as described previously [2]. Protein samples were separated on 12% v/v SDS-PAGE, transferred onto NC membrane (Millipore, Billerica, MA, USA), and then subsequently blocked with TBST (5% BSA) at room temperature for 1 h, followed by incubation at 4 °C overnight with appropriate antibodies. After the samples were incubated with horseradish peroxidase-conjugated secondary antibody for 1 h at room temperature, the specific protein bands were visualized by enhanced chemiluminescence (ECL) substrate (Beyotime, Shanghai, China). The results were quantified by densitometry using ImageJ software (version 1.53) and the densitometry results were normalized relative to the β-actin bands.

#### 4.4.5. Detection of ROS Generation in RAW264.7 Cells

The original medium was removed, and DCFH-DA was diluted according to the instructions of the ROS test kit. The DCFH-DA was added away from light, incubated in a 37 °C cell incubator for 30 min, and washed for three times. Finally, the intracellular fluorescence intensity was observed by inverted fluorescence microscope and quantified by ImageJ software.

### 4.5. CCL_4_-Induced Acute Liver Injury and Allicin Pretreatment

#### 4.5.1. Animal Experimental Design

Male Balb/c mice aged 6−7 weeks and weighing 18−22 g were procured from Jiangsu Jicui Yaokang Biotechnology Co., Ltd. (Nanjing, China). They were housed in a controlled environment with a temperature of 23 ± 2 °C, relative humidity of 50 ± 10%, and a 12-h light–dark cycle. Prior to experimentation, all mice underwent a one-week acclimatization period and were provided ad libitum access to food and water throughout the study.

Mice were randomly divided into 7 groups (6 mice/group) as the following: normal control (NC); allicin group (40 mg/kg); CCL_4_ group (CCL_4_); CCl_4_^+^Allicin 40 mg/kg, CCl_4_^+^Allicin 20 mg/kg, and CCl_4_^+^Allicin 10 mg/kg; and CCl_4_^+^compound glycyrrhizin group 80 mg/kg (CCl_4_^+^CGI80). The mice in the control and allicin groups were administrated with an equal volume of vehicle or allicin (40 mg/kg). In the CCl_4_ group, mice were intraperitoneally (i.p.) injected with 1% CCl_4_ (dissolved in peanut oil, 10 mL/kg). In the CCl_4_^+^Allicin 40 mg/kg, CCl_4_^+^Allicin 20 mg/kg, and CCl_4_^+^Allicin 10 mg/kg groups, mice were given allicin prior to being intraperitoneally injected with 1% CCl_4_, and then continued to be given allicin every 12 h by intragastric administration. The treatment of CCl_4_^+^CGI80 group is the same with CCl_4_^+^Allicin groups. Following 24 h of hepatotoxicity caused by CCl_4_, the mice in each group were weighed and sacrificed after being anesthetized with sevoflurane; blood and liver tissue were immediately collected. The livers were weighed, washed with pre-cooled saline, and divided into samples. One part was fixed in 4% paraformaldehyde for histopathological examination, while the remainder was immediately frozen in liquid nitrogen for subsequent experiments. The serum was extracted by centrifuging the blood at 3000 rpm for 10 min at 4 °C, and immediately subjected to biochemical analysis.

#### 4.5.2. Hepatic Histological Detection

The liver lobes from each group, fixed in 10% formalin, were embedded in paraffin and sectioned into 4 µm thick slices for subsequent H&E staining. All sections were examined and photographed using an optical microscope.

#### 4.5.3. Liver Function Evaluation

The levels of serum transaminase (ALT) and aspartate transaminase (AST), which are critical indicators of hepatocellular injury, were measured by using an Automated Chemical Analyzer (Mindray BS-240VET, Mindray Biomedical Electronics Co., Ltd., Shenzhen, China) with the standard diagnostic kits (Mindray Biomedical Electronics Co., Ltd., Shenzhen, China).

#### 4.5.4. Assay of Malondialdehyde (MDA) Activities and Catalase (CAT) Activities

The liver was homogenized in nine volumes of cold phosphate buffer (pH 7.4). The supernatants were separated at 10,000 rpm for 10 min at 4 °C and used to assay the activities MDA and CAT with a commercially available assay kit (Solarbio, Beijing, China) according to the method described.

#### 4.5.5. Cytokine Measurement Using Enzyme Linked Immunosorbent Assay (ELISA)

The levels of TNF-α, IL-6, and IL-1β in serum were analyzed by the ELISA kits according to the manufacturer’s instructions (Proteintech, Wuhan, China), respectively.

#### 4.5.6. Western Blot Analysis of Related Proteins in Liver Tissue

The ground liver tissue was placed in pre-cooled cracking solution and homogenized in a multi-sample tissue homogenizer at a frequency of 60 Hz for 20 s, and placed on ice for 5 min to ensure complete cracking. After centrifugation at high speed at 4 °C and 12,000 r/min for 20 min, the supernatant was taken to obtain the total protein. After the protein concentration was determined by BCA method, the protein concentration was 4 mg/mL, 12% SDS-PAGE was prepared, the sample size was 10 μL; and the protein was transferred to NC membrane by the semi-dry conversion method, enclosed in 5% (g/mL) defatted milk for 2 h at room temperature, and incubated overnight at 4 °C with the primary antibody. TBST was washed and reacted with the secondary antibody for 2 h. After washing, ECL luminescent solution was added and exposed.

### 4.6. Statistical Analysis

All data are presented as mean ± SD. The statistical analyses were performed using SPSS V11.5. A one-way analysis of variance (ANOVA; Pb0.05) was used to determine significant differences between groups and the individual comparisons were obtained by Tukey’s HSD (honestly significant difference) post hoc test. A *p*-value < 0.05 was considered as statistical significance.

## Figures and Tables

**Table 1:** Gene primer sequence used in qPCR.

Numbers	Genes	Sequence (5’−3’)	Product Length
NM_008361.4	IL-1β	F: GGGCCTCAAAGGAAAGAATC	183
		R: TACCAGTTGGGGAACTCTGC	
NM_031168.2	IL-6	F: AGTTGCCTTCTTGGGACTGA	191
		R: CAGAATTGCCATTGCACAAC	
NM_011198.5	Cox-2	F: AGAAGGAAATGGCTGCAGAA	194
		R: GCTCGGCTTCCAGTATTGAG	
NM_007393.5	β-actin	F: CCACAGCTGAGAGGGAAATC	193
		R: AAGGAAGGCTGGAAAAGAGC	

## Data Availability

All data are available in the manuscript.
